# Health- related quality of life and self-worth in 10-year old children with congenital hypothyroidism diagnosed by neonatal screening

**DOI:** 10.1186/1753-2000-6-32

**Published:** 2012-10-03

**Authors:** Liesbeth van der Sluijs Veer, Marlies JE Kempers, Heleen Maurice-Stam, Bob F Last, Tom Vulsma, Martha A Grootenhuis

**Affiliations:** 1 Pediatric Psychosocial Department, Emma Children’s Hospital AMC, A3-241, P.O. Box 22700,, 1100,, DE, Amsterdam, The Netherlands; 2 Department of Pediatric Endocrinology, Emma Children’s Hospital, Academic Medical Center, Amsterdam, The Netherlands; 3 Department of Clinical Genetics, Radboud Universtiy Nijmegen Medical Centre, Nijmegen, The Netherlands; 4 Department of Developmental psychology, VU University, Amsterdam, The Netherlands

**Keywords:** Congenital hypothyroidism, Quality of life, Self-worth, Children

## Abstract

**Background:**

Much is written about cognitive and motor development; less is known about social and emotional consequences of growing up with congenital hypothyroidism (CH).

The objectives of the study were: (1) to compare health related quality of life (HRQoL) and self-worth of 10 year old patients with CH with the general population; (2) to explore associations of disease factors, IQ and motor skills with the outcomes.

**Methods:**

Children with CH and their parents completed several questionnaires. Patients were classified to ‘severe CH, n = 41’ or ‘moderate/mild CH, n = 41’ based on pre-treatment FT4 concentration.

Differences between CH and the general population were tested by analysis of covariance and one sample t-tests (mean scale scores HRQoL and self-worth), chi-square tests and binomial tests (% at risk of impaired HRQoL and self-worth). Linear regression analyses corrected for gender were conducted to explore associations of the outcomes with disease factors, IQ and motor skills.

**Results:**

Patients with CH reported lower mean HRQoL on motor, cognitive and social functioning, and on autonomy and positive emotions (p < 0.0001). Patients were also more often at risk for impaired HRQoL and self-worth. No differences were found between the severity groups. Lower IQ was only significant associated with worse cognitive HRQoL. Initial FT4 plasma, age at onset of therapy, initial T4 dose and motor skills were not significantly associated with HRQoL and self-worth.

**Conclusions:**

Negative consequences in terms of HRQoL and self-worth are prevalent in children with CH, independent of disease factors, IQ and motor skills. Physicians should to be attentive to these consequences and provide attention and supportive care.

## Background

Severe intellectual disability associated with congenital hypothyroidism (CH) is prevented by newborn screening and early treatment. However, children with CH still undergo a brief period of thyroid hormone deficiency reflecting etiology of thyroid disease, severity and treatment factors. Thyroid hormone is essential for almost all life processes, but most important for normal development of the central nervous system of the fetus and the infant. Neonatal screening programmes for CH have been effective in preventing serious cognitive and motor deficits through early initiation of T4 supplementation [1,2]. However, several studies showed that children and adults with CH, especially those with severe CH, still experience a range of cognitive and motor deficits [3-6].

Clearly, much is written about cognitive and motor development of children with CH. There is a growing body of literature directed at social and emotional consequences of children growing up with CH. Many psychological studies conclude that children with different chronic diseases are at higher risk for emotional and behavior problems [7,8]. Some studies found behavior disorders and psychiatric disturbances in children with CH [8-11]. Other studies were directed at assessing health related quality of life (HRQoL) in young adults with CH [12-14]. However, HRQoL and self-worth in children have not been studied thoroughly. HRQoL can be used as an indicator of adjustment, which covers the patient’s perceptions of his or her physical, emotional, social and cognitive functions, as well as the patient’s perceived health status and well-being [15]. In addition, positive self-worth is a significant factor influencing overall mental health and psychological well-being [16,17], and is regarded by major theorists as a basic psychological need [18].

CH is a chronic life-long disease [19], which may affect the patient’s daily life because of the hospital visits, the daily T4 administration, the need of regular dose adjustments and sometimes the need of adjuvant medical care such as speech training and physiotherapy. Besides, CH could have a negative impact on motor skills and in some (severely affected) patients also on the cognitive development [3], which in turn might affect their social life, self-esteem and emotional functioning.

In order to be able to adequately support the psychomotor development of children with CH, insight in their social-emotional functioning is necessary. Therefore the purpose of the present study was (1) to assess HRQoL and self-worth in children with CH at ten years of age born in 1992–1993 and compare the results to those of the general (healthy) population, and (2) to explore the influence of disease factors, IQ and motor skills on HRQoL and self-worth.

## Methods

### Screening method and treatment strategy

The Dutch neonatal CH screening method is primarily based on the measurement of T4 in filter paper blood spots. In 1992 and 1993 sampling was performed between 5 and 8 days after birth. T4, expressed as standard deviation score, is compared to the day mean. If T4 was ≤ −0.8 SD, thyrotropin (TSH) was additionally measured. When T4 was ≤ −3.0 SD or TSH was ≥50 μU/ml children were referred immediately. Children with a dubious result (−3.0 < T4 ≤ −2.1 SD, or 25 ≤ TSH < 50 μU/ml) underwent a second heelpuncture and were referred if the result was again dubious, or abnormal. The etiological classification of CH was based upon initial presentation, thyroid function determinants and thyroid imaging.

In 1992–93 Dutch pediatricians were advised to start with T4-supplementation in a dose of 6 to 8 μg/kg.day. In accordance with international guidelines T4-dose adjustments were based on thyroid function determinants, obtained at regular outpatient follow-up visits.

### Sample

The complete cohort of patients with CH born in The Netherlands in 1992 and 1993 consisted of 141 patients (Table 1). Patients were classified as CH-T (CH of thyroidal origin), CH of central origin (CH-C) or CH not yet specified. CH-T was further classified as CH-T due to thyroid agenesis, thyroid dysgenesis, or thyroid dyshormogenesis. In this study, only children with CH-T were included.

**Table 1:** Characteristics of the 1992–1993 cohort of patients with congenital hypothyroidism (CH)

**Etiology**	**Total**	**Non-participants**		**Participants**
		**not suitable **	**not willing**	
CH-T due to thyroid agenesis	24	6	1	17
CH-T due to thyroid dysgenesis	51	2	1	48
CH-T due to thyroid dyshormonogenesis	24	3	4	17
CH-C	16	15	1	0
CH n.o.s.	26	15	11	0
Total CH	141	41	18	82

Four groups are presented; the total group, the group of patients who did not participate divided in patients not suitable or not willing to participate and the group of patients who did participate. For each group, the subdivision according to etiological classification is given. CH n.o.s., CH not otherwise specified.

From the original cohort 3 patients had died, 4 had moved abroad and 4 had transient CH. The parents of the remaining 130 patients were contacted via their pediatricians, whose responses led to the exclusion of patients with CH-C (n = 15), with a known or suspected syndrome (n **=** 9), with a brain tumour (n = 1), and patients of whom the mother was treated with T4 during pregnancy (n = 2). Three patients were excluded because the recommended dose adjustments were not made in time due to misunderstandings (Table 1, ‘not suitable’ to participate). Furthermore, the parents of 18 patients declined participation (Table 1, ‘not willing’ to participate). Parents of a total of 82 patients gave their written informed consent. To ascertain that the participating patients were well-treated (i.e. TSH 0.4-4.0 μU/ml) at the time of testing, the most recent measurement of thyroid function prior to the psychological tests was evaluated and if necessary T4-dose was adjusted. This resulted in dose adjustments for 20 patients. Patients were classified to subgroups based on their pre-treatment FT4 concentration: ‘severe CH’: FT4 ≤ 0.3 ng/dL (≤4 pmol/L), ‘moderate/mild CH’: FT4 > 0.3 ng/dL (FT4 > 4.0 pmol/L). The reference range for FT4 is 0.9-2.2 ng/dL (12–28 pmol/L) for children aged 2–6 weeks.

### Procedure

All children and their parents were asked to complete the questionnaires in the Academic Medical Center (AMC) (except for 7 patients who were tested in their local hospitals) under the supervision of the same psychologist (LvdSV), who was blinded for the patients’ medical details. The assistance of the psychologist was restricted to explaining the meaning of difficult words. The study protocol was approved by the institutional review board of the Emma Children’s Hospital/Academic Medical Center and the privacy committee of the Dutch CH Screening Board.

### Measures

#### HRQoL

Health-related quality of life (HRQoL) of the children with CH was assessed with the TNO-AZL Children’s Quality of Life questionnaire; Parent Form for children aged 6 to 11 years (TACQoL-PF) [20] and Child Form for children aged 8 to 15 years (TACQoL-CF) [21,22]. These questionnaires are originally Dutch instruments that measure generic HRQoL [20-23]. The questionnaires measure health status problems weighted by the impact of the health status problems on well-being. It offers the respondent the possibility of differentiating between their functioning and the way they feel about it. The items are clustered into multi-item scales with higher scores indicating better quality of life. The TACQoL (CF and PF) contains seven scales of eight items each: physical functioning, autonomy, motor functioning, cognitive functioning and school performances, social functioning, positive emotions and negative emotions. The Cronbach’s alphas in our study population were moderate to good (0.67-0.87) with the exception of the Autonomy scale of the TACQoL-CF (Cronbach’s alpha <0.4). Age-matched norm data from the Dutch general population were available. Children with a chronic medical condition in this norm population were excluded. This resulted in a norm population of 449 healthy children. The psychometric properties, validity and reliability, of the TACQoL are satisfactory [20-23].

#### Self-worth

The translated version of the The Self-Perception Profile for Children (CBSK) [24] was used to assess patients’ self-worth. The CBSK, meant for children aged 8–12 years, consisted of 36 items. Each answer was scored between 1 (most competent) and 4 (least competent). Several aspects of self-perception were measured in six subscales, each consisting of six items: school competence, social acceptance, athletic competence, physical appearance, behavioral conduct and global self-worth. The Cronbach’s alphas of the CBSK scales in our study population were moderate to good (0.67-0.78). Dutch norms are based on a representative sample of 361 Dutch children. The psychometric properties and reliability of the CBSK are satisfactory [24].

#### Intelligence (IQ) and motor skills

Intelligence was measured with the Dutch version of the Wechsler Intelligence Scale for Children, 3^rd^ Edition, (WISC-III) [25]. Three intelligence quotients were derived: Full Scale Intelligence Quotient (FSIQ); Verbal Intelligence Quotient (VIQ); and Performance Intelligence Quotient (PIQ). In the normative population, each IQ-score has a mean of 100 (SD 15).

Motor skills were assessed with the Movement Assessment Battery for Children (MABC) [26,27], designed for identification of impairments of motor function in children aged 4–12 years. The test results are expressed in terms of a total motor impairment score (‘Total MABC score’); higher scores indicate more motor problems.

For a comprehensive description of these measures we refer to our study on IQ and motor outcome of ten year old patients with CH [3].

### Statistical analysis

Data were analysed using SPSS version 12.0 (SPSS Inc., Chicago, IL). Before conducting the final analyses several preparation analyses were conducted. Firstly, scale scores were computed and missing data imputed on the basis of the guidelines of the TACQoL [17]. In calculation of the scale scores, one missing combined-item score was allowed for. The missing score was replaced by the mean value of the non-missing item scores. Second, the internal consistencies (Cronbach’s alphas) of the scales were calculated, and the distributions of the scale scores were considered.

HRQoL and self-worth of the group with CH were compared with that of the norm population in two ways: (1) using mean scale scores, (2) using the percentage of children at risk of impaired HRQoL and self-worth. Patients with CH (total group) were compared to the norm population, and thereafter the severe and moderate/mild subgroups as well. Differences between the severe and moderate/mild subgroups with CH were also tested. Analyses of covariance (ANCOVA) were conducted to test for differences on the TACQoL mean scale-scores between the group with CH and norm population and between the severity groups, corrected for gender. Because the database of the CBSK norm data was not available, we used the mean scores that were presented in the manual of the CBSK. Therefore one sample t-tests were performed to test whether the several CBSK-scales scores (means) of the children with CH differed from the means in the norm population [24]. Patients with CH (total group) were compared to the norm population, and thereafter the severe and moderate/mild subgroups as well. Differences between the severity groups regarding the CBSK-scale scores were tested with ANCOVA. To adjust for multiple testing, we used a Bonferroni correction and adjusted the alpha to 0.007 (0.05/7) for the TACQoL and 0.008 (0.05/6) for the CBSK. Effect sizes (*d*) were calculated by dividing the difference in mean score between the patients with CH (total group) and norm population by the standard deviation of the scores in the norm population. We considered effect sizes up to 0.2, 0.5, and 0.8 to be small, moderate and large respectively [28].

To create a clinically meaningful distinction, children with CH are categorized into being ‘at risk’ or ‘not at risk’ for problems (i.e. impaired HRQoL, impaired self-worth), based on percentile norms by age and gender in the norm population. The value of the 25 ^th^ percentile in the norm population was used for the scales of the TACQoL. Although there is no gold standard for good or bad HRQoL yet, this definition is considered to be a suitable way to differentiate between individuals with higher scale scores from individuals with lower scale scores [29]. The percentage at risk in the group with CH was compared with the percentage in the norm population using Chi-square tests (χ^2^-tests, p < 0.007;0.05/7). The definition of being at risk for impaired self-worth was based on the value of the 15 ^th^ percentile (CBSK scales) in the norm population as recommended in the manual of the CBSK [24]. We tested whether the percentage of children with CH with scores below the value of the 15^th^ percentile in the norm population was different from 15% using binomial tests (p < 0.008; 0.05/6).

Linear regression analyses were conducted to explore the influence of disease factors (initial FT4 plasma, age at onset therapy, initial T4 dose) on HRQoL and self-worth. Regression models were also fitted for HRQoL and self-worth predicted by full scale IQ, and predicted by motor skills (total score). All regression models were corrected for gender. A significance level of 0.007 (0.05/7) was used for the HRQoL scales and 0.008 (0.05/6) for the scales of self-worth. Because of the explorative nature of the regression analyses, regression coefficients at p < 0.05 and p < 0.01 were also reported, to be considered as a trend.

## Results

### Sample characteristics

The characteristics of the participating patients with CH and their parents are given in Table 2. Of the 82 patients with CH (53 girls, 65%), 41 had severe CH and 41 had moderate/mild CH. The median age at start of T4 supplementation was 20 days for the total group. The IQ scores of the participating patients with CH are given in Table 2. For all details, we refer to a previous publication [3].

**Table 2:** Characteristics of participating CH patients and their parents

**PATIENTS**	**Severe CH**	**Moderate/Mild CH**	
*Number of patients* (male/female)	41 (13/28)	41 (16/25)	
*Initial FT4*			
in ng/dl (95%CI)*	0.1 (0.0-0.3)	0.7 (0.3-1.1)	
[in nmol/l (95%CI)]	[1.8 (0.0-4.0)]	[9.4 (4.2-20.2]	
*Age at start of T4 supplementation* in mean days (range)	19 (10–43)	25 (2–73)	
Mean IQ scores at 10.5 yr **	**Severe**	**Moderate**	**Mild**
Full scale IQ	93.7 (89.5-97.9)	96.2 (88.9-103.5)	105.0 (99.5-110.4)
Verbal IQ	94.9 (90.1-99.7)	95.4 (87.9-102.9)	103.6 (98.2-109.1)
Performance IQ	93.9 (90.0-97.8)	98.0 (91.1-104.9)	105.3 (99.3-111.3)
Mean motor scores at 10.5 yr **			
Total MABC score***	14.3 (11.8-16.8)	9.7 (6.8-12.5)	11.6 (8.7-14.6)
**PARENTS**			
*Number of the participating parents* (mothers/fathers)	35 (26/9)	38 (33/5)	
*Parental marital status*			
Married/living together, n (%)	32 (91)	37 (97)	
Single, n (%)	3 (9)	1 (3)	
*Educational level father*			
Low, n (%)	11 (31)	18 (47)	
Middle, n (%)	11 (31)	11 (29)	
High, n (%)	13 (38)	9 (24)	
*Educational level father*			
Low, n (%)	17 (47)	18 (47)	
Middle, n (%)	12 (34)	12 (30)	
High, n (%)	7 (19)	8 (23)	

* Reference range for FT4 in children aged 2–6 wk is 0.9-2.2 ng/dlL (12–28 pmol/l) (34).

** *published in Kempers et al. (3).*

*** No motor problems: (Total MABC score ≤ 9.5), borderline motor problems : (9.5 < Total MABC score < 13.5, definite motor problems: (Total MABC score ≥ 13.5).

### Health related quality of life (HRQoL): mean scores

#### Child-report

The total group with CH reported significantly worse HRQoL than the norm population on four out of the seven scales of the TACQoL: motor functioning F(1,524) = 18.04; cognitive functioning F(1,523) = 20.52; social functioning, F(1,521) = 13.47; positive emotions F(1,522) = 16.10. The differences were small to moderate; effect sizes (d) ranged from 0.3 to 0.6 (Table 3).

**Table 3:** **Health-related quality of life (TACQoL)**^**1**^**: patients with congenital hypothyroidism (CH) versus the norm population; Mean scores, Standard deviations (SD’s) and effect sizes**^**2**^

	**Total CH**	**Severe CH**	**Moderate/Mild CH**	**Norm population**	**Effect size**
	**Mean (SD)**	**Mean (SD)**	**Mean (SD)**	**Mean (SD)**	**Total**
**Child-report**					
	n = 82	n = 41	n = 41	n = 449	
*Physical Functioning*	23.7 (4.8)	23.3 (4.9)	24.0 (4.6)	25.2 (5.0)	0.3
*Motor Functioning*	28.3 (3.5) ***	27.9 (3.8) ***	28.7 (3.3) *	29.8 (3.1)	0.5
*Autonomy*	30.9 (1.5)	30.5 (1.7) **	31.3 (1.1)	31.5 (1.5)	0.4
*Cognitive functioning*	26.5 (4.9) ***	25.3 (5.1) ***	27.7 (4.4)	28.7 (3.7)	0.6
*Social Functioning*	28.6 (4.0) ***	28.0 (4.2) ***	29.1 (3.9)	29.9 (2.5)	0.5
*Positive emotions*	12.6 (2.6) ***	13.0 (2.4)	12.2 (2.8) ***	13.7 (2.4)	0.5
*Negative emotions*	12.0 (2.5)	11.4 (2.6)	12.5 (2.3)	11.9 (2.6)	0.0
**Parent-report**					
	n = 82	n = 41	n = 41	n = 465	
*Physical Functioning*	26.9 (4.4)	26.5 (4.3)	27.3 (4.3)	27.3 (3.8)	0.1
*Motor Functioning*	29.3 (2.8) ***	28.7 (3.2) ***	30.0 (2.3)	30.8 (2.3)	0.7
*Autonomy*	31.2 (1.9)	31.1 (1.9)	31.2 (2.0)	31.6 (1.2)	0.3
*Cognitive functioning*	26.0 (5.2) ***	25.1 (5.7) ***	26.9 (4.5) **	29.0 (3.7)	0.8
*Social Functioning*	29.0 (3.1) **	28.9 (3.0)	29.1 (3.3)	29.9 (2.5)	0.4
*Positive emotions*	14.5 (2.1)	14.4 (2.3)	14.7 (2.0)	14.7 (2.0)	0.1
*Negative emotions*	11.0 (2.4) **	10.9 (1.8)	11.2 (2.8)	11.8 (2.4)	0.3

^1^Higher scores represent better HRQoL: range 0 – 32 for physical, motor, autonomy, cognitive, social; range 0 – 16 for positive and negative moods.

^2^effect size (d): total CH versus norm population.

*p < 0.007: difference between CH patients and norm population according to ANOVA by group and gender.

**p < 0.001: difference between CH patients and norm population according to ANOVA by group and gender.

***p < 0.0001: difference between CH patients and norm population according to ANOVA by group and gender.

HRQoL of the *severe* group with CH appeared to be significantly worse than that of the norm population on four scales: motor functioning F(1,483) = 14.74; autonomy F*(*1,483) = 11.774*;* cognitive functioning F(1,483) = 28.07; social functioning F(1,482) = 16.40, while the *moderate/mild* group with CH scored worse than the norm on motor functioning and positive emotions; F(1,487) = 6.62 and F(1,486) = 15.589 respectively.

#### Parent-report

Parents of patients with CH (total group) reported significantly worse HRQoL in their children than parents of the norm population on four out of the seven scales of the TACQoL: motor functioning F(1,543) = 23.76; cognitive functioning F(1,543) = 38.18; social functioning F(1,543) = 8.21; negative emotions F(1,543) = 6.77. The differences were small to large; effect sizes (d) ranged from 0.4 to 0.8 (Table 3).

HRQoL of the *severe* group with CH appeared to be significantly worse (p < 0.007) than that of the norm population on motor and cognitive functioning; F(1,502) = 26.61 and F(1,502) = 36.36 respectively. The *moderate/mild* group with CH scored only significantly worse than the norm on cognitive functioning F(1,503) = 11.61.

### Self-worth: mean scores

With respect to the self-worth only one significant difference was found (Table 4). The girls with CH reported lower social acceptance than the girls in the norm population; T = (1,50) = −2.75, effect sizes (d) = 0.4.

**Table 4:** **Self-worth (CBSK)**^**1**^**: patients with congenital hypothyroidism (CH) versus the norm population; Mean scores, Standard deviations (SD’s) and effect sizes**^**2**^

	**Total CH**	**Severe CH**	**Moderate/Mild CH**	**Norm group**	**Effect size**
	**Mean (SD)**	**Mean (SD)**	**Mean (SD)**	**Mean (SD)**	**Total**
**Boys**					
	n = 27	n = 11	n = 16	n = 180	
School competence	17.2 (3.2)	16.4 (3.7)	17.7 (2.8)	17.4 (3.5)	0.1
Social acceptance	18.6 (3.8)	17.8 (4.7)	19.2 (3.0)	17.8 (3.8)	0.2
Athletic competence	17.6 (3.8)	16.6 (4.4)	18.3 (3.2)	18.7 (3.3)	0.3
Physical appearance	20.0 (2.9)	19.4 (3.0)	20.4 (2.9)	20.1 (3.6)	0.0
Behavioral conduct	17.6 (3.1)	17.6 (3.3)	17.6 (2.6)	17.0 (2.8)	0.2
General self-worth	20.0 (2.7)	19.9 (3.1)	20.0 (2.5)	20.0 (3.0)	0.0
**Girls**					
	n = 51	n = 26	n = 25	n = 181	
School competence	15.4 (3.6)	15.8 (3.4)	14.9 (3.8)	16.3 (3.4)	0.3
Social acceptance	15.9 (4.0) ^*^	16.2 (4.0)	15.7 (3.3)	17.5 (3.7)	0.4
Athletic competence	16.1 (3.9)	17.0 (4.1)	16.5 (3.7)	17.6 (3.8)	0.4
Physical appearance	18.5 (4.2)	18.6 (4.5)	18.4 (4.0)	18.9 (4.0)	0.1
Behavioral conduct	17.0 (3.5)	17.5 (3.3)	16.5 (3.3)	18.0 (3.4)	0.3
General self-worth	19.1 (2.6)	19.5 (3.7)	18.6 (3.0)	19.4 (3.5)	0.1

^1^Higher scores represent better self-worth: range 6 – 24 for all scales.

^2^effect size (d): total CH versus norm population.

* p < 0.007: difference between CH patients and norm population according to one-sample *t*-test.

### Differences according to CH severity (severe versus mild/moderate): mean scores HRQoL and Self-worth

There were no significant differences found between the severity groups for HRQoL and self-worth (Table 3 and 4).

### Impaired HRQoL: percentage at risk

#### Child-report

The total group of patients with CH showed significantly higher percentages of children at risk for impaired HRQoL (38% to 48%) than the norm population on four scales of the TACQoL: motor functioning, autonomy, cognitive functioning and positive emotions (Table 5).

**Table 5:** Percentage at risk for impaired health related quality of life (TACQoL): patients with congenital hypothyroidism (CH) versus the norm population

	**Total CH**	**Severe CH**	**Moderate/Mild CH**	**Norm population**
**Child-report**				
	n = 82	n = 41	n = 41	n = 449
*Physical Functioning*	40%	46% *	34%	25%
*Motor Functioning*	48% **	50% *	46% *	25%
*Autonomy*	38% **	46% **	29%	25%
*Cognitive functioning*	40% *	57% **	25%	25%
*Social Functioning*	34%	41%	28%	25%
*Positive emotions*	45% *	42%	47% *	25%
*Negative emotions*	30%	36%	25%	25%
**Parent-report**				
	n = 82	n = 41	n = 41	n = 465
*Physical Functioning*	34%	37%	32%	25%
*Motor Functioning*	55% **	61% **	49%*	25%
*Autonomy*	27%	32%	22%	25%
*Cognitive functioning*	56% **	61% **	51%*	25%
*Social Functioning*	29%	34%	24%	25%
*Positive emotions*	34%	37%	25%	25%
*Negative emotions*	38%	39%	37%	25%

^1^ 25 ^th^ Percentiles of norm population are not exact 25% due to distribution of scale scores; percentiles approach 25^th^, ranging from 17^th^ – 31^st^ percentile.

*p < 0.007: difference between CH patients and norm population according to Chi-square test **p < 0.0001: difference between CH patients and norm population according to Chi-square test.

In the *severe* group, significantly more patients with CH than children in the norm population were considered at risk for problems with physical, motor and cognitive functioning, and with autonomy; ranging from 46% to 57%. Patients in the *moderate/mild* group with CH were considered more at risk for problems with motor functioning (46%) and positive emotions (47%).

#### Parent-report

According to the parents, patients with CH (total, severe and moderate/mild) had a significantly greater risk for impaired HRQoL than children in the norm population on two scales of the TACQoL: motor and cognitive functioning: 49% and 61% respectively (Table 5).

### Impaired Self-worth: percentage at risk

Patients with CH (total, severe, moderate/mild) showed higher percentages at risk for impaired self-worth than the norm population with regard to school competence and athletic competence; ranging from 27 to 34% versus 15% in the norm group (Table 6).

**Table 6:** Percentage at risk for impaired Self-worth (CBSK): patients with congenital hypothyroidism (CH) versus the norm population

	**Total CH**	**Severe CH**	**Moderate/Mild CH**	**Norm group**
	**n = 82**	**n = 41**	**n = 41**	**n = 361**
School competence	30% *	32% *	27%*	15%
Social acceptance	24%	24%	24%	15%
Athletic competence	33% *	32% *	34% *	15%
Physical appearance	15%	14%	17%	15%
Behavioral conduct	26%	22%	29%	15%
General self-worth	19%	22%	17%	15%

*p < 0.008: difference between CH patients and norm population according to binominal test.

### Asssociations of disease factors, IQ and motor skills with HRQoL and self-worth

The regression analyses demonstrated only one significant result: higher full scale IQ was found to be correlated with better (parent reported) cognitive functioning (β = 0.38; p = 0.001).

Several trends could be reported, based on significance levels of 0.01 and 0.05. Regarding the disease factors, higher initial T4 dose was associated with better (parent-reported) physical HRQoL (β = 0.37; p < 0.05). Furthermore, higher full scale IQ was found to be correlated with better HRQoL regarding (child reported) negative emotions (β = 0.28; p < 0.05) and (parent reported) positive emotions (β = 0.23; p < 0.05). Finally, better motor skills were associated with higher (parent reported) HRQoL scores on motor functioning (β = 0.24; p < 0.05) and autonomy (β = 0.30; p < 0.01), and with better self-worth; social acceptance (β = 0.24; p < 0.05) and athletic competence (β = 0.29; p < 0.05).

## Discussion

This is one of the first studies that assessed self-worth and health related quality of life (HRQoL) using a self- and proxy report in early treated children with CH. The results of the study showed that CH could have a negative impact on several aspects of HRQoL and self-worth. Ten year old children with CH born in 1992–1993 experienced worse HRQoL than the norm population with respect to cognitive -, motor- and social functioning, positive emotions, negative emotions and autonomy. In addition, a greater percentage of children with CH, especially patients with severe CH, appeared to be at risk for impaired HRQoL as well as for impaired self-worth with respect to school performance and athletic performance.

These results are in line with our previous results among young adults with CH, who reported also lower HRQoL and lower self-worth than healthy peers [12]. Two other studies evaluated the HRQoL in young adults with CH [13,14]. Sato et al. showed that the HRQoL of young adults did not differ from healthy controls [13]. In a recent study of Leger et al. [14], young adults with CH had a lower HRQoL than their healthy peers, as in our study. These authors underlined the need of early and consequent monitoring patients with CH **[**[14]. Several other studies showed that children and adolescents with CH were at risk of social-emotional problems, such as behavioral disorders and psychiatric disturbances. Tinelli et al. [30] found that adolescents (> 12 years) scored significantly higher than controls on withdrawal, anxiety/depression, thought problems, attention problems and aggressive behavior. Bisacchi et al. [14] found more internalizing and externalizing problems in 6–10 years patients with CH. However, they found no differences between patients and controls in other age groups. The results of our study can be considered in line with a growing body of literature about the psychological and social consequences of medical treatment in children with chronic diseases. Many of these studies concluded that children with a chronic disease show more maladjustment than healthy children [7,8,31].

The most persuasive result is that patients with CH reported considerably worse parent- and child-reported HRQoL in the domains “motor functioning” and “cognitive functioning”, also presented in a high percentage of patients considered at risk for impaired HRQoL in these domains. In addition, children with CH appeared to be more at risk for low self-worth in school competence and athletic competence. We also found that lower IQ was associated with lower scores on cognitive functioning and that worse motor skills tended to be associated with worse self-worth regarding athletic competence. These findings are important and require further elaboration. The lower scores on cognitive and motor functioning we found in our study are in line with the outcomes of diverse neuropsychological studies, in which was found that children and young adults with CH scored significantly lower than the norm population on motor functioning [3,5,6,28-30] and had more problems with attention and memory [32-36]. So, the patient’s perception of motor and cognitive functioning, as measured with the TACQoL, equates with objective findings. However, worse IQ and motor skills did not explain the presence of impaired functioning in most other domains of HRQoL and self-worth, as the results of the regression analyses demonstrated. So, we can conclude that patients with CH are at risk for impaired HRQoL and self-worth, independent of their IQ and motor skills. Furthermore, no significant association of severity, intial T4 dose and age at onset of therapy with HRQoL and self-worth was found.

Therefore, it could assume that living with a chronic disease as such and/or the negative consequences of CH despite of its severity, influence functioning in daily life. CH affects the child’s daily life because of the need of regular T4-dose adjustments, the daily T4 administration, frequent T4 and TSH measurements, consciousness of having a chronic disease, and sometimes the need of adjuvant medical care such as speech training and physiotherapy. In addition, the cognitive and motor problems of patients with CH may affect their social life, self-worth and emotional functioning. From this study and our previous studies [3,9,37], it is apparent that patients with CH seem to be vulnerable in these areas**.** Besides, one has to keep in mind that a suboptimal thyroid hormone state may affect well-being. Whereas the goal of long-term T4 treatment is to maintain euthyroidism, this remains challenging because of the continuous need to adapt T4 dose in a growing child and the need of treatment compliance. It has been shown that differences in serum FT4 and TSH concentrations, even within the reference range, may be determinants of psychological well-being in treated hypothyroid patients [38].

The strength of our study is that we tested a nationwide cohort of patients with CH, all treated by pediatricians who followed national guidelines, and that at psychological assessment, all patients had plasma TSH concentrations within the reference range. Besides, HRQoL was assessed by self-report as well as by the parents of the children with CH. Moreover, we tried to strengthen the clinical meaning of the results, by using percentages at risk of impaired HRQoL and self-worth as outcomes, in addition to mean scale scores. This is considered a suitable way because a golden standard for bad HRQoL and self-worth is lacking.

The limitations of the current study should also be taken into account. First, the loss of subjects from the original cohort restricts the representativeness of the current sample. However, we clarified the etiology of both the excluded patients and the patients not willing to participate (Table 1). Second, we could not use a control group and no information about the socio- economic status of the norm population was available. In general however, Dutch normative data of standardized measures such as the CBSK and TACQoL are sufficient to make adequate comparisons.

Third, caution is called for generalizing our results to children who are nowadays growing up with CH because treatment protocols changed since the 1992–1993. The question is, whether the impaired HRQoL of patients with CH in our study could be assigned to suboptimal treatment years ago, as lower initial T4 dose and older age at onset of therapy compared to the current treatment protocols. In the present we did not found any significant association of initial T4 dose and age at onset of therapy with HRQoL and self-worth, which might be an indication that the contribution of treatment factors to psychosocial outcomes in patients with CH is limited. So, it could be assumed that also children with CH being treated nowadays should be considered at risk of impaired HRQoL and self-worth.

Another shortcoming of the study is that we did not examine other potential risk- and protective factors of HRQoL and self-worth, as socio-economic and psychosocial factors (e.g. parenting, family functioning, coping). Future research should be directed at these factors, in order to be able to detect and support the children and adolescents who are at risk for impaired psychosocial functioning at an early stage. Finally, because of the cognitive and motor problems in patients with CH, it seems important to examine the effect of adjuvant care like physiotherapy, speech training and intervention programs directed at the improvement of cognitive functions.

### Conclusion and Clinical implications

This study has shown that children with CH, diagnosed by neonatal screening, are at increased risk for impaired quality of life and self worth. In particular, our findings add to the evidence for motor and cognitive problems in relation to CH. Following this, we can conclude that children with CH are vulnerable and that there is need for specific care. We believe that these results deserve proper attention and awareness of physicians treating these children. Furthermore, patients with CH and their parents should become more aware of the possible negative consequences of growing up with CH. Follow-up of patients with CH should not only be a medical/biochemical evaluation but, also to attain the best achievable quality of life. The focus during the follow-up should shift to attention to school performances, social-emotional functioning and supporting the patients. Therefore routine monitoring HRQoL and social-emotional functioning in children with CH is recommended. Incorporating patient reported outcomes of HRQoL in daily clinical practice will contribute to better communication with health care professionals and makes it easier for them to refer to the needed care if necessary [39,40]. In addition, the use of valid and reliable screening instruments to detect patients with CH at risk for social, emotional and behavior problems are recommended, for example the Strength and Difficulties Questionnaire (SDQ) [41]. When motor problems are present, patients should be motivated to engage in sport activities or should be referred to the physiotherapist if needed. When cognitive problems are present, psychological examination would be useful and if necessary, intervention programs that improve cognitive functions such as memory and attention functioning or speech training might thereafter be offered to particular individuals. Finally, it seems important to stimulate children’s social performance and to support children with their social skills.

## Abbreviations

HRQoL: Health-related quality of life; CH: Congenital hypothyroidism.

## Competing interests

The authors declare that they have no competing interests.

## Authors’ contribution

LvdSV conceptualized and designed the study, collected the data, carried out the analyses, drafted the initial manuscript, and approved the final manuscript as submitted. MJEK conceptualized and designed the study, collected the data, reviewed and revised the manuscript, and approved the final manuscript as submitted. HMS critically reviewed the analyses, reviewed and revised the manuscript, and approved the final manuscript as submitted. TV conceptualized and designed the study and approved the final manuscript as submitted. BFL conceptualized and designed the study, reviewed and revised the manuscript and approved the final manuscript as submitted. MMG conceptualized and designed the study, reviewed and revised the manuscript, and approved the final manuscript as submitted. All authors participated in the design of the study. LVDSV drafted the manuscript. MJEK, HMS, BFL TV and MAG edited the manuscript. All authors read and approved the final manuscript.

## Acknowledgement

This study is supported by a grant (22000144) from The Netherlands Organization for Health Research and Development (ZON-MW), The Hague,The Netherlands.
